# The COVID-19 Pandemic and Antibiotic Use on the United States–Mexico Border

**DOI:** 10.1017/ash.2021.89

**Published:** 2021-07-29

**Authors:** Sana Khan, Katherine Ellingson, Gemma Parra, Juan Villanueva, Carlos Garrido

## Abstract

**Background:** The US–Mexico border represents a unique region of the country where antibiotics are more accessible and nonprescription treatment with antibiotics is deeply enculturated. Currently, both the United States and Mexico are experiencing widespread community transmission of SARS-CoV-2, which may have implications for antibiotic seeking and use. The objective of this study was to examine antibiotic seeking behavior as it relates to COVID-19 in the border region relative to the greater US and Mexico populations. **Methods:** An interdisciplinary team at The University of Arizona developed a survey to assess knowledge, attitudes, and beliefs about antibiotics along the US–Mexico border region (defined as 100 km from the border) and to compare findings from the border region to the broader US and Mexico populations. The team recruited survey participants through Amazon’s MTurk survey platform and through the distribution of recruitment flyers to community partners in Arizona and Mexico border regions from October 2020 to January 2021. Targeted recruitment was 750 through March 2021. We report here on findings from the first round of recruitment (n = 116). These participants were asked whether they had sought out antibiotics specifically as a treatment for COVID-19, as well as their general beliefs and behaviors on self-seeking antibiotics for illness. **Results:** As of January 24, 2021, we surveyed 116 participants: 82 (70.7%) from the United States and 34 (29.3%) from Mexico. Most participants (71.2%) were aged 25–44 years; 56.9% were male; and 50% reported Hispanic ethnicity. Of these, 13.8% lived within 100 km of the US–Mexico border. Overall, 21.6% of participants reported taking antibiotics to fight COVID-19–like illness. Of these participants, 28% obtained the antibiotics directly from a pharmacy, without a physician prescription, and 16% obtained them from an online vendor. Additionally, 33% of US respondents reported that they would be willing to travel to Mexico to obtain antibiotics if they were too difficult to obtain in the United States. Of these respondents, 55% said they would be willing to travel for >1 hour to obtain antibiotics. **Conclusions:** Preliminary data suggest that the COVID-19 pandemic will have widespread ramifications on antibiotic seeking behavior and could propagate antibiotic resistance. Targeted intervention strategies in the border region are necessary to mitigate the unique factors that contribute to antibiotic use in this area.

**Funding:** No

**Disclosures:** None

